# The impact of illness perception on parenting concerns of breast cancer patients with minor children: mediating effects of fear of progression

**DOI:** 10.3389/fpsyg.2025.1676894

**Published:** 2025-12-11

**Authors:** Wantian Liang, Yi Pang, Jiejie Lin, Jingjing Liu, Wenli Liang

**Affiliations:** 1 School of Nursing, Guangdong Pharmaceutical University, Guangzhou, China; 2 Department of Continuing Education, Guangdong Pharmaceutical University, Guangzhou, China

**Keywords:** breast cancer, fear of progression, illness perception, mediating effect, minor children, parenting concerns

## Abstract

**Objective:**

To examine the impact of illness perception on parenting concerns among breast cancer patients with minor children, specifically focusing on the mediating effects of fear of progression.

**Methods:**

A convenience sample of 315 female breast cancer patients with minor children were selected from the Breast Surgery Clinic at a tertiary women’s and children’s hospital in Guangzhou city between September 2023 and December 2024. Participants were surveyed using a general information questionnaire, Parenting Concerns Questionnaire (PCQ), The Brief Illness Perception Questionnaire (B-IPQ), and Fear of Progression Questionnaire-Short Form (FoP-Q-SF). Multiple linear regression was applied to identify factors influencing parenting concerns, and structural equation modeling was used to examine the mediating role of fear of progression in the relationship between illness perception and parenting concerns.

**Results:**

The mean score for parenting concerns of breast cancer patients with minor children was 2.28 ± 0.83, illness perception mean score was 40.14 ± 11.51, and fear of progression mean score was 34.50 ± 8.74. Correlation analysis suggested that parenting concerns were positively correlated with both illness perception and fear of progression (*p* < 0.05). Multiple linear regression analysis indicated that the age group of the youngest child, illness perception, and fear of progression were independent factors influencing parenting concerns. Moreover, women who viewed their illness more negatively also reported more parenting concerns, and about 18.18% of this relationship was explained by fear of progression.

**Conclusion:**

Patients with breast cancer generally experience varying degrees of parenting concerns for their minor children. Illness perception directly predicts parenting concerns, and also indirectly predicts them through the mediating effects of fear of progression. Healthcare providers should pay attention to the parenting concerns of breast cancer patients with minor children and use proactive psychological interventions and comprehensive support systems to correct negative disease perceptions, reduce fear of progression, and effectively alleviate parenting concerns, thereby improving psychological adaptation and family functioning.

## 1 Introduction

Breast cancer (BC) is one of the leading causes of cancer-related mortality among women worldwide, often referred to as the “pink killer” (Lei et al., 2021). 2022 data from the International Agency for Research on Cancer indicates that approximately 2.3 million new breast cancer cases were reported globally, accounting for 11.6% of all new cancer cases and making it the second most prevalent cancer worldwide (Bray et al., 2024). In China, approximately 357,000 new breast cancer cases were diagnosed in 2022. This accounted for 23.8% of all female cancers, making it the second most common cancer after lung cancer (Han et al., 2024). Notably, breast cancer rates have been rising annually, with a marked trend toward younger onset in recent years (He et al., 2024). A growing number of young patients with breast cancer face the dual challenge of undergoing treatment while raising minor children (Li et al., 2024). Studies indicate that approximately 30% of women initially diagnosed with breast cancer have at least one minor child (Chinese Society of Clinical Oncology, 2023). Compared to patients without children or those with adult children, mothers of minors experience significantly greater parenting-related stress and poorer overall quality of life (Inhestern et al., 2016a). The intersection of life-threatening illness and childcare responsibilities creates pervasive parenting concerns among patients with breast cancer (Lundquist et al., 2020).

Parenting concerns, broadly defined, refer to the anxiety and worries that parents experience regarding the upbringing of their children (Schmitt et al., 2008). Hymovich (1993) first introduced the concept of parenting concerns in patients with cancer, defining it as the emotional stress arising from the difficulty of balancing parenting with the challenges of managing the disease. Parenting concerns are especially common in patients with breast cancer. In Asian cultures, women often carry the main responsibility for raising children, which can increase stress during illness (Frese and Nguyen, 2022). A mixed-methods study revealed that mothers with breast cancer prioritize their parental identity over their patient identity (Zhang et al., 2025). However, treatment-related physical side effects often impair parenting capacity, reduce parent–child interaction, and diminish maternal role satisfaction, thereby exacerbating parenting concerns (Lundquist et al., 2020). Research has shown that excessive parenting concerns not only contributes to anxiety and depression (Park et al., 2016), but may also spread through emotional contagion within the family, weakening family emotional bonds and adversely affecting children’s psychological development (Daundasekara et al., 2021). Negative emotions can weaken the immune system, potentially reducing the body’s ability to respond to treatment (Rahal et al., 2023). Therefore, investigating the determinants and mechanisms that influence parenting concerns in breast cancer patients with minor children is of great significance.

Leventhal’s Common-Sense Model of Self-Regulation posits that an individual’s cognitive appraisal of their illness significantly influences their coping strategies and emotional responses (Leventhal et al., 2016). Illness perception, a central concept in this model, refers to an individual’s subjective understanding and interpretation of their disease, including their views on its causes, consequences, duration, and controllability (Buse et al., 2023). The more negative the illness perception, the more likely patients are to experience strong negative emotions and psychological stress (Lee et al., 2023). Previous studies have confirmed that illness perception is a significant predictor of parenting concerns in patients with breast cancer, with a positive correlation between the two (Li et al., 2025). A qualitative study shown that for patients with breast cancer raising minor children, a negative perception of their illness increases concerns about not being able to accompany their children through life’s milestones, which worsens parenting concerns; conversely, a more positive perception of the illness, with the belief that it is controllable and treatable, correlates with lower levels of parenting concerns (Rashi et al., 2015).

Moreover, illness perception has been identified as a key predictor of Fear of Progression (FoP) in patients with breast cancer. The stronger the negative perception of their illness, the higher their fear of progression (Safdari-Molan et al., 2023). Fear of Progression, defined as the fear of the perceived potential for disease progression or recurrence, and the associated negative physiological, psychological, and social consequences (Herschbach and Dinkel, 2014), ranks among the most prevalent yet under-addressed psychosocial concerns in cancer survivors (Chen et al., 2020). A study by Reed et al. (2020) involving 1,032 patients with cancer found that 73% of patients with breast cancer reported varying degrees of fear of progression. Research based on a cross-sectional survey indicates that fear of progression can persist for up to 16 years after diagnosis, significantly impairing the quality of life for cancer survivors (Koch-Gallenkamp et al., 2016). The uncertainty surrounding disease progression causes ongoing psychological distress, which may trigger negative emotional reactions like anxiety and depression (Lucas et al., 2023). For breast cancer patients with minor children, fear of progression may amplify concerns about their ability to fulfill parenting responsibilities, further worsening parenting concerns (Romare Strandh et al., 2025). A study by Arès et al. (2014) further confirmed that the greater the fear of progression, the higher level of parenting concerns.

Although previous studies have explored the relationships between illness perception, fear of progression, and parenting concerns, most have been limited to pairwise correlation analyses, and the path relationships among these three variables remain unclear. Given that illness perception correlates with parenting concerns (Li et al., 2025) and fear of progression correlates with both illness perception (Safdari-Molan et al., 2023) and parenting concerns (Arès et al., 2014), it is possible that fear of progression serves as a mediator between illness perception and parenting concerns. Moreover, most studies on parenting concerns have focused on the general cancer population (Strandh et al., 2024), with limited attention given to patients with breast cancer who are raising minor children. In particular, within Asian cultural contexts, the role of mother is often seen as a vital component of female self-identity and societal responsibility, suggesting that parenting concerns in this group may carry stronger emotional and cultural specificity (Zhang et al., 2025).

This study explores how illness perception and fear of progression relate to parenting concerns in Chinese patients with breast cancer raising children under 18. A mediation model will be developed to test the mediating role of fear of progression in the relationship between illness perception and parenting concerns. The goal is to elucidate the underlying mechanisms and provide empirical evidence for developing targeted intervention strategies to improve the psychological health of these patients and promote minor children well-being.

## 2 Methods

### 2.1 Study design and sample

We recruited patients with breast cancer from three campuses of a tertiary hospital in Guangzhou using convenience sampling. Patients were enrolled during inpatient or outpatient visits between September 2023 and December 2024. Inclusion criteria were: (1) pathological diagnosis of primary breast cancer, clinical stage I–IV; (2) age ≥ 20 years; (3) at least one child under the age of 18; (4) awareness of their own condition and voluntary participation in the study. Exclusion criteria were: patients with severe physical illnesses or mental disorders. This study complies with the ethical principles of the Declaration of Helsinki and was approved by the Ethics Committee of Guangzhou Women and Children’s Medical Center ([2023]196A01).

We used the semPower package in R to calculate the required sample size (Jobst et al., 2023) based on 3 latent and 8 observed variables. With *α* = 0.05 and 95% power, the target was 256 participants. Accounting for a 20% invalid response rate, the final goal was 308; the study included 315.

### 2.2 Measurements

#### 2.2.1 General information questionnaire

This questionnaire was self-designed by the researchers and consists of two parts: (1) demographic data (age, marital status, education level, occupation, per capita monthly household income, payment system of medical expenses, number of children, number of minor children, and the age group of the youngest child). (2) disease-related data (disease duration, cancer stage, type of surgery, chemotherapy, radiotherapy, family history of cancer, and history of chronic disease).

#### 2.2.2 The parenting concerns questionnaire (PCQ)

This questionnaire was developed by Muriel et al. (2012) and translated into Chinese by Kang et al. (2021). It consists of 15 items across 3 dimensions: concerns about the emotional impact on children, concerns about the practical impact on children, and concerns about the co-parent. It uses a 5-point Likert scale, ranging from “not concerned” to “extremely concerned” (scored 1–5). The total score and dimensional scores are calculated by averaging the item scores, with higher scores indicating greater parenting concerns. According to Toothaker’s classification rule (Toothaker, 1994), scores below one standard deviation from the mean are classified as mild levels of parenting concerns, scores above one standard deviation as higher levels of parenting concerns, and scores in between as moderate levels of parenting concerns. In this study, the Cronbach’s *α* coefficient for this questionnaire was 0.945.

#### 2.2.3 The brief illness perception questionnaire (B-IPQ)

This questionnaire was developed by Broadbent et al. (2006) and translated into Chinese by Mei et al. (2015). It contains 9 items across 3 dimensions: cognition, emotion, and comprehension. The first 8 items are scored on a scale from 0 to 10 (with reverse scoring for items 3, 4, and 7), and item 9 is an open-ended question for attribution only and no score. The total score ranges from 0 to 80, with higher scores indicating a greater burden of illness perception. In this study, the Cronbach’s *α* coefficient for this questionnaire was 0.739.

#### 2.2.4 The fear of progression questionnaire-short form (FoP-Q-SF)

This questionnaire was simplified from the Fear of Progression Questionnaire by Mehnert et al. (2006), and the Chinese version was translated by Wu et al. (2015). It consists of 12 items across 2 dimensions: physiological health and social family. A 5-point Likert scale is used, ranging from “never” to “always” (scored 1–5). The total score ranges from 12 to 60, with higher scores indicating greater fear of progression. Scores of 12–23 are classified as mild fear, 24–36 as moderate fear, and 37–60 as severe fear. In this study, the Cronbach’s α coefficient for this questionnaire was 0.906.

### 2.3 Data collection

The researchers explained the purpose and significance of the study to the participants using standardized instructions. After obtaining informed consent, paper questionnaires were distributed. The questionnaire completion time was controlled at 15 to 20 min. The questionnaires were completed and collected on-site, and any missing responses were promptly addressed. To minimize the risk of response bias, data collectors only reminded participants to fill in missing responses without offering suggestions or influencing their answers, ensuring participants’ autonomy and response neutrality. After the participants completed the surveys, the researcher collected disease-related data by reviewing electronic medical records. A total of 328 questionnaires were distributed, and 315 were effectively collected, with an effective response rate of 96.04%.

### 2.4 Statistical analysis

We analyzed data using SPSS 26.0. Frequencies and percentages were reported for categorical variables; means and SDs for continuous ones. We used *t*-tests and ANOVAs with Bonferroni *post hoc* tests, Pearson correlations, and stepwise multiple regression. There were no missing values in the dataset. Harman’s one-way test was used to examine common method bias, and AMOS 23.0 software was used to construct a structural equation model of illness perception, fear of progression, and parenting concerns. The maximum likelihood estimator was used in AMOS. The Bootstrap method was used to verify direct and indirect effects of each path. Significance was set at *α* = 0.05.

## 3 Results

### 3.1 Characteristics of participants

A total of 315 patients with breast cancer were included in the study, with ages ranging from 29 to 56 years, and a mean age of 42.15 ± 5.19 years. Among these patients, 205 (65.1%) were under 45 years old, 309 (98.1%) were married, and 123 (39.0%) had a bachelor’s degree and above. The majority were company employees (119, 37.8%), and 119 (37.8%) reported a monthly household income per capita between 5,000 and 9,999 RMB. For payment system of medical expenses, 252 (80.0%) used resident-based medical insurance. Regarding family structure, 199 (63.2%) patients had more than two children, 134 (42.5%) had more than two children under 18 years old, and 132 (41.9%) had the youngest child in the school-age range. In terms of disease-related data, 165 patients (52.4%) had a disease duration of more than 12 months. Cancer staging showed that 147 patients (46.7%) were at stage I. Surgical treatment involved 141 patients (44.8%) undergoing radical mastectomy. Regarding treatment history, 216 patients (68.6%) had a history of chemotherapy, 92 (29.2%) had a history of radiotherapy, 45 (14.3%) had a family history of cancer, and 40 patients (12.7%) had a history of chronic diseases. The demographic and disease-related characteristics of the patients with breast cancer are shown in Table 1.

**Table 1:** Demographic and disease-related data on parenting concerns of breast cancer patients with minor children (*N* = 315).

Variables	Group	*N* (%)	Parenting concerns (*M* ± SD)	*t/F*	*p*
Age (years)	20 ~ 45	205 (65.1)	2.23 ± 0.83	−1.676^a^	0.095
	45 ~ 59	110 (34.9)	2.39 ± 0.81		
Marital status	Married	309 (98.1)	2.29 ± 0.82	0.716^a^	0.474
	Divorced	6 (1.9)	2.04 ± 1.05		
Education level	Primary school and below	30 (9.5)	2.26 ± 0.79	0.500^b^	0.736
	Junior high school	23 (7.3)	2.48 ± 1.12		
	Senior high school or technical secondary school	64 (20.3)	2.22 ± 0.77		
	Junior college	75 (23.8)	2.32 ± 0.73		
	Bachelor’s degree and above	123 (39.0)	2.26 ± 0.86		
Occupation	Company employee	119 (37.8)	2.32 ± 0.88	0.185^b^	0.906
	Administrative sector employee	47 (14.9)	2.28 ± 0.84		
	Freelancer	87 (27.6)	2.27 ± 0.78		
	Unemployed	62 (19.7)	2.23 ± 0.79		
Per capita monthly household income (yuan)	<3,000	20 (6.3)	2.41 ± 1.06	2.236^b^	0.084
	3,000 ~ 4,999	79 (25.1)	2.40 ± 0.76		
	5,000 ~ 9,999	119 (37.8)	2.32 ± 0.79		
	≥10,000	97 (30.8)	2.11 ± 0.85		
Payment system of medical expenses	Government-sponsored medical insurance	31 (9.8)	2.23 ± 0.86	1.505^b^	0.224
	Resident-based medical insurance	252 (80.0)	2.26 ± 0.79		
	Self-paying	32 (10.2)	2.52 ± 1.04		
Number of children	1	116 (36.8)	2.18 ± 0.80	−1.665^a^	0.097
	≥2	199 (63.2)	2.34 ± 0.84		
Number of minor children	1	181 (57.5)	2.23 ± 0.82	−1.345^a^	0.180
	≥2	134 (42.5)	2.36 ± 0.83		
Age group of the youngest child	Infancy and Toddlerhood	26 (8.3)	1.93 ± 0.65	2.671^b^	0.048
	Preschool age	62 (19.7)	2.22 ± 0.87		
	School-age	132 (41.9)	2.40 ± 0.79		
	Adolescence	95 (30.2)	2.26 ± 0.87		
Disease duration (months)	<3	92 (29.2)	2.36 ± 0.82	0.959^b^	0.413
	3 ~ <6	28 (8.9)	2.08 ± 0.77		
	6 ~ <12	30 (9.5)	2.36 ± 0.91		
	≥12	165 (52.4)	2.26 ± 0.82		
Cancer stage	I	147 (46.7)	2.27 ± 0.78	0.139^b^	0.937
	II	111 (35.2)	2.32 ± 0.87		
	III	48 (15.2)	2.25 ± 0.84		
	IV	9 (2.9)	2.24 ± 1.09		
Type of surgery	No	12 (3.8)	2.58 ± 1.12	0.967^b^	0.408
	Radical mastectomy	141 (44.8)	2.24 ± 0.82		
	Breast-conserving surgery	118 (37.5)	2.34 ± 0.83		
	Radical mastectomy + reconstruction	44 (14.0)	2.20 ± 0.76		
Chemotherapy	Yes	216 (68.6)	2.27 ± 0.85	−0.325^a^	0.746
	No	99 (31.4)	2.31 ± 0.78		
Radiotherapy	Yes	92 (29.2)	2.35 ± 0.85	0.970^a^	0.333
	No	223 (70.8)	2.26 ± 0.82		
Family history of cancer	Yes	45 (14.3)	2.30 ± 0.82	0.133^a^	0.894
	No	270 (85.7)	2.28 ± 0.83		
History of chronic disease	Yes	40 (12.7)	2.48 ± 0.90	1.623^a^	0.106
	No	275 (87.3)	2.26 ± 0.81		

a *T*-tests.

b One-way ANOVA.

There was a statistically significant difference in parenting concern scores based on the age group of the youngest child (*F* = 2.671, *p* = 0.048). Pairwise comparisons revealed that patients with breast cancer whose youngest child was in the school-age range had significantly higher parenting concern scores than those whose youngest child was in the infancy and toddlerhood. No other demographic and disease-related characteristics showed significant differences (*p* > 0.05).

### 3.2 Mean scores, and correlations between variables

In this study, the mean score for parenting concerns among the 315 patients with breast cancer was 2.28 ± 0.83. Among them, 49 patients (15.6%) had mild levels of parenting concerns, 210 (66.7%) had moderate levels of parenting concerns, and 56 (17.8%) had higher levels of parenting concerns. The illness perception mean score was 40.14 ± 11.51, and the mean score for fear of progression was 34.50 ± 8.74. The scores for each variable are shown in Table 2. Pearson correlation analysis revealed that parenting concerns in patients with breast cancer were positively correlated with illness perception (*r* = 0.458, *p* < 0.01) and fear of progression (*r* = 0.370, *p* < 0.01). Additionally, illness perception was positively correlated with fear of progression (*r* = 0.285, *p* < 0.01). The detailed results are presented in Table 3.

**Table 2:** Mean scores for illness perception, fear of progression, and parenting concerns (*N* = 315).

Variables	*M* ± SD
Illness perception	40.14 ± 11.51
Comprehension	3.30 ± 2.39
Emotion	13.74 ± 3.97
Cognition	23.10 ± 8.02
Fear of progression	34.50 ± 8.74
Physiological health	18.03 ± 4.30
Social family	16.47 ± 5.02
Parenting concerns	2.28 ± 0.83
Concerns about the emotional impact on children	2.39 ± 0.93
Concerns about the practical impact on children	2.35 ± 0.89
Concerns about the co-parent	2.12 ± 0.89

**Table 3:** Correlation analysis between illness perception, fear of progression and parenting concerns (*N* = 315).

Variables	Correlation analysis										
	1	2	3	4	5	6	7	8	9	10	11
1. Illness perception	1										
2. Comprehension	0.514**	1									
3. Emotion	0.681**	0.050	1								
4. Cognition	0.944**	0.415**	0.468**	1							
5. Fear of progression	0.285**	0.080	0.268**	0.252**	1						
6. Physiological health	0.238**	0.062	0.238**	0.206**	0.928**	1					
7. Social family	0.292**	0.087	0.263**	0.263**	0.948**	0.761**	1				
8. Parenting concerns	0.458**	0.291**	0.279**	0.433**	0.370**	0.287**	0.399**	1			
9. Concerns about the emotional impact on children	0.417**	0.256**	0.288**	0.379**	0.333**	0.256**	0.361**	0.937**	1		
10. Concerns about the practical impact on children	0.464**	0.255**	0.310**	0.437**	0.401**	0.319**	0.425**	0.939**	0.871**	1	
11. Concerns about the co-parent	0.380**	0.289**	0.167**	0.377**	0.284**	0.216**	0.311**	0.875**	0.700**	0.713**	1

**p < 0.01.

### 3.3 Multiple linear regression analysis of parenting concerns

Multiple stepwise regression analysis was performed with the parenting concerns scores as the dependent variable, and illness perception, fear of progression, and statistically significant variables from the univariate analysis as independent variables. The results indicated that illness perception, fear of progression, and the age group of the youngest child were significant predictors and entered the regression equation. All regression coefficients reported in this section are standardized estimates to facilitate interpretation of the relative strength of associations. The detailed results are presented in Table 4.

**Table 4:** Multiple linear regression analysis for parenting concerns using standardized coefficients (*N* = 315).

Model	*B*	*SE*	*β*	*t*	*p*	*95%CI*	
						LLCI	ULCI
Constant	0.051	0.234	–	0.219	0.827	−0.408	0.511
Illness perception	0.027	0.004	0.378	7.520	<0.001	0.020	0.034
Fear of progression	0.026	0.005	0.271	5.377	<0.001	0.016	0.035
Age group of the youngest child	0.088	0.044	0.097	2.011	0.045	0.002	0.175

All coefficients reported in this table are standardized estimates (*β*). *R*^2^ = 0.282; adjusted *R*^2^ = 0.275; *F* = 40.685; *p* < 0.001.

### 3.4 Analysis of the mediating effect of fear of progression between illness perception and parenting concerns

Harman’s one-way test revealed that a total of 6 common factors with eigenvalues > 1 were extracted, with the first factor explaining 31.92% of the variance, which is below the 40% critical threshold (Tang and Wen, 2020). Therefore, this study does not exhibit significant common method bias.

**Figure 1:**
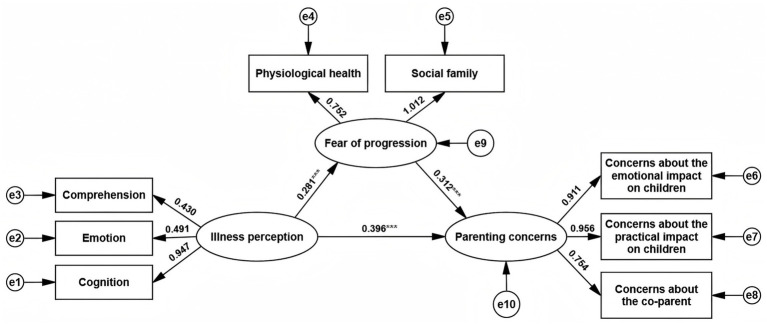
The mediation model of illness perception, fear of progression and parenting concerns.

A structural equation model was constructed with illness perception as the independent variable, fear of progression as the mediator, and parenting concerns as the dependent variable. All path coefficients in the model were standardized, as shown in Figure 1. The model fit indices, including NC, GFI, AGFI, NFI, IFI, CFI, and RMSEA, all met the standard criteria for model fit, indicating that the model fits the data well, as summarized in Table 5.

**Table 5:** Fit indices and reference standards of the model (*N* = 315).

Fit indices	Reference standards	Outcome	Judgment
NC (*χ*^2^/df)	1 < NC < 3	2.824	Yes
GFI	>0.9	0.963	Yes
AGFI	>0.9	0.921	Yes
NFI	>0.9	0.962	Yes
IFI	>0.9	0.975	Yes
CFI	>0.9	0.975	Yes
RMSEA	<0.08	0.076	Yes

Using the Bootstrap method, 5,000 resamples were drawn from the data, and the 95% confidence intervals (*95% CI*) for the effect sizes were calculated. If the confidence interval does not include zero, the effect is considered significant. The results showed that women who viewed their illness more negatively also reported more parenting concerns, and about 18.18% of this relationship was explained by fear of progression, as detailed in Table 6.

**Table 6:** The mediating effect of fear of progression between illness perception and parenting concerns (*N* = 315).

Effect	Estimate	BootSE	*95%CI*		Effect size (%)	*p*
			LLCI	ULCI		
Total effect Illness perception → parenting concerns	0.484	0.078	0.312	0.622	100%	0.001
Direct effect Illness perception → parenting concerns	0.396	0.077	0.238	0.545	81.82%	<0.001
Indirect effect Illness perception → fear of progression → parenting concerns	0.088	0.025	0.045	0.146	18.18%	<0.001

## 4 Discussion

This study revealed that patients with breast cancer commonly exhibited varying degrees of parenting concerns, with a mean score of 2.28 ± 0.83. Compared to the study by Inhestern et al. (2016b) 1.76 ± 0.76, this study found higher scores, which may be attributed to various factors, including cultural differences, as Eastern societies often emphasize familial roles and responsibilities, which may contribute to heightened concerns related to parenting among breast cancer patients (Li et al., 2025). These cultural dynamics could influence the psychological burden and coping strategies of individuals facing both cancer treatment and family responsibilities. In this study, 84.5% of patients with breast cancer reported moderate to higher levels of parenting concerns, indicating significant psychological stress related to their responsibilities for raising their minor children. This highlights the need for greater attention from both clinical and social perspectives. Additionally, the study found significant differences in parenting concerns among breast cancer patients with children in different age groups. Specifically, patients with children in the school-age range had higher parenting concern scores than those with children in infancy or toddlerhood. This could be due to the greater dependency for maternal presence during the academic, psychological, and social development of school-age children (Salerni and Messetti, 2025). When patients are unable to fulfill their maternal duties due to illness, they may experience a profound sense of role loss and guilt, believing they are failing in their parental responsibilities, which exacerbates their parenting concerns (Li et al., 2024). However, current healthcare services often focus primarily on cancer treatment and tend to overlook the psychological needs of patients in terms of parenting (Kim et al., 2024). Medical institutions should collaborate with community, family, and other resources to provide psychological and parenting support, helping patients rebuild their role identity and alleviate parenting concerns.

This study also showed that the illness perception score for patients with breast cancer was 40.14 ± 11.51, which was higher than the findings of Liu et al. (2022). This difference may be related to age characteristics, as 65.1% of the participants in this study were young patients with breast cancer. This group is in a critical life stage, balancing the early stages of their career, raising children, and caring for elderly parents. A breast cancer diagnosis disrupts their life plans, making their perception of the disease’s threat and negative impact more intense. The fear of progression in these patients had a mean score of 34.50 ± 8.74, indicating moderate fear, which is consistent with the study by Zhuang et al. (2024). This result suggests that fear of progression has become a prevalent psychological condition among patients with breast cancer.

Correlation analysis revealed that parenting concerns were positively correlated with illness perception. The higher the level of illness perception, the greater degree of parenting concerns, which aligns with previous research (Li et al., 2025). This could be because illness perception reflects the patient’s subjective understanding of their condition. The more negative their perception of breast cancer, the more likely they are to worry about their health prognosis and their family’s well-being, fearing they may not be able to accompany and care for their children long-term, which increases parenting concerns. Additionally, parenting concerns were positively correlated with fear of progression, with a higher level of fear correlating with greater parenting concerns, consistent with previous studies (Arès et al., 2014). When patients excessively fear disease progression, they may develop negative expectations about the future, projecting their anxiety onto their children, excessively worrying about their children’s future and quality of life, which intensifies parenting concerns (Faraji et al., 2023). Illness perception was also positively correlated with fear of progression. When patients hold negative views about their condition, they are more likely to worry about disease deterioration, recurrence, or metastasis, leading to sustained fear.

Mediation analysis revealed that women who viewed their illness more negatively also reported more parenting concerns, and about 18.18% of this relationship was explained by fear of progression. This suggests that illness perception not only directly predicts parenting concerns in patients with breast cancer but also indirectly influences them through fear of progression. While the mediation effect was not dominant, it indicates that fear of progression is a key factor connecting illness perception and parenting concerns. During the diagnosis and treatment process, patients with breast cancer often experience uncertainty about their condition, particularly regarding prognosis and recurrence risk (Son et al., 2023). When patients have a pessimistic view of their disease and lack clear, positive information, they are more likely to develop catastrophic thinking, intensifying their fear of progression (Wang et al., 2025). This fear leads patients to focus their concerns on their relationship with their children, particularly their ability to care for and spend time with them, which exacerbates parenting concerns. Therefore, in clinical practice, helping patients develop a clearer understanding of their illness and alleviating their excessive fear of progression could be an important intervention to reduce the transformation of fear into parenting concerns. Cognitive Behavioral Therapy (CBT) is a widely used psychological intervention for patients with cancer. By guiding patients to identify and reframe irrational beliefs about their illness, CBT helps them understand their health status from a more positive perspective, alleviating the negative emotional reactions triggered by illness perception (Ekekezie et al., 2023). Future interventions, such as CBT, might help reduce fear of progression by reshaping illness beliefs—but this needs to be tested in longitudinal or experimental studies. Park’s study of 74 non-metastatic patients with breast cancer in Japan showed that mindfulness-based CBT, which helped patients recognize their negative cognitive patterns and foster a more positive mindset, significantly improved their overall well-being, including psychological, physical, and spiritual aspects (Park et al., 2020). Therefore, future clinical interventions should consider incorporating CBT into the comprehensive psychological care plan for patients with breast cancer, as an important strategy to alleviate their parenting concerns and improve family functioning.

## 5 Limitations

This study reveals the pathway mechanisms between illness perception, fear of progression, and parenting concerns among breast cancer patients with minor children. However, there are several limitations. First, the study uses a cross-sectional design, which can only capture associations between variables at a single point in time. This limits the ability to explore the dynamic changes in the psychological states of patients with breast cancer throughout the disease and treatment stages, thereby restricting a deeper understanding of the causal relationships between variables. Future research could consider conducting longitudinal studies to track the evolution of patients’ psychological states over time and further explore the relationship between illness perception, fear of progression, and parenting concerns. Second, the sample in this study was drawn from three campuses of a tertiary women’s and children’s hospital in Guangzhou city, China. This results in a relatively homogenous sample in terms of geographic location and medical background, and does not include patients from grassroots medical institutions or community follow-ups, which could affect the generalizability of the findings. Future studies should consider including multiple regions and a broader range of medical institutions to increase the representativeness of the sample and enhance the generalizability and applicability of the results. Additionally, this study only examined fear of progression as a mediating variable between illness perception and parenting concerns, without considering other potential psychosocial factors. Future research could include additional relevant variables to provide a more comprehensive understanding of the factors influencing parenting concerns among patients with breast cancer. Finally, although Harman’s single-factor test was conducted to assess common method bias, this approach has limitations, as it assumes that method bias manifests in a single dominant factor. More robust alternatives could be considered in future research.

## 6 Conclusion

Patients with breast cancer generally experience varying degrees of parenting concerns for their minor children, with higher degrees of parenting concerns observed in those whose youngest child was in the school-age range. Illness perception can directly influence parenting concerns, but can also have an indirect effect through the mediation of fear of progression. These findings suggest that healthcare professionals should pay close attention to breast cancer patients’ subjective perceptions of the disease and their emotional responses, particularly their fear of progression. Strengthening disease education and providing psychological support, which can be delivered through evidence-based interventions such as CBT, can help patients better understand their condition and alleviate fears about disease progression. Additionally, the psychological needs of patients in their role as mothers should not be overlooked. It is recommended that a multi-layered collaborative support system be developed, involving national, hospital, community, and family-level efforts to alleviate parenting concerns, enhance psychological adaptation, and improve family functioning.

## Data Availability

The original contributions presented in the study are included in the article/Supplementary material, further inquiries can be directed to the corresponding author.
